# Useful Items

**Published:** 1873-08

**Authors:** 


### USEFUL ITEMS.

Sixty drops, one teaspoonful, or drachm.
Four teaspoonfuls, one tablespoonful.
Four tablespoonfuls, one ounce.
Sixteen ounces, one pint, or pound.
Four ounces, one gill.
Two gills make half a pint.
Two pints make one quart.
A common tumbler holds half a pint.
One wine-glass is half a gill.
A teacup is one gill.

### WEIGHTS AND MEASURES.

Wheat flour, one pound is one quart.
Indian meal, one pound two ounces are one quart.
Butter, when soft, one pound is one quart.
Loaf sugar, broken, one pound is one quart.

White sugar, powdered, one pound one ounce are one quart.

Best brown sugar, one pound two ounces are one quart.

Ten eggs are one pound.

Flour, eight quarts are one peck.

Flour, four pecks are one bushel.

Sixty grains make one drachm, eight drachms make one ounce, twelve ounces make a pound, in medicine.

